# Post‐Finasteride Syndrome or Pre‐Existing Vulnerability? Rethinking Patient Selection

**DOI:** 10.1111/jocd.70243

**Published:** 2025-05-14

**Authors:** Nicolò Rivetti

**Affiliations:** ^1^ Dermatology Outpatient Clinic Istituto Clinico Beato Matteo, Gruppo San Donato Vigevano Pavia Italy

**Keywords:** androgenetic alopecia, psychological aspects, trichology

Post‐finasteride syndrome (PFS) refers to a constellation of persistent physical, sexual, and neuropsychiatric symptoms that have been reported by some individuals following the discontinuation of finasteride, a 5α‐reductase inhibitor commonly used for androgenetic alopecia. Despite proper counseling, I regularly see patients who discontinue finasteride within weeks—not due to pharmacologic intolerance, but out of fear triggered by online testimonials. The discourse surrounding PFS has heightened awareness of persistent adverse effects associated with finasteride use. While the etiology of PFS remains under investigation, an often overlooked aspect is the psychological profile of patients prior to initiating finasteride therapy [1, 2, 3].

The phenomenon of nocebo responses is well‐documented in medical literature, where negative expectations can lead to the onset or worsening of symptoms—even in the absence of a pharmacological cause [1]. This effect may be particularly pronounced in patients undergoing aesthetic or hair loss treatments, who are often hyper‐focused on physical changes and intensely monitor their bodily sensations. In such individuals, a single alarming testimony encountered online can trigger anxiety and lead to the emergence of symptoms like fatigue or decreased libido—driven more by fear than by pharmacologic action. This highlights the importance of addressing psychological factors when prescribing medications like finasteride, which have been associated with both real and perceived adverse effects [2, 3, 4, 5]. Physicians may also play a key role in countering misinformation by proactively discussing common online claims, recommending evidence‐based resources, and encouraging patients to bring their doubts into the consultation room rather than relying on unverified testimonials. Additionally, science‐based communication by healthcare professionals on social media can help balance the online narrative and reduce the influence of alarmist content on vulnerable individuals [6]. This pattern of obsessive monitoring and cyberchondria can further reinforce a nocebo response, compounding the patient's fear and accelerating treatment discontinuation. These traits are commonly associated with health anxiety and somatic symptom disorders, and may be interpreted within the cognitive‐behavioral model, where negative expectations and misinterpretation of bodily sensations contribute to symptom amplification [4].

In many such cases, what is later labeled as “post‐finasteride syndrome” may in fact reflect a pre‐existing cognitive vulnerability reacting to a widely stigmatized medication [1, 4]. Rather than debating whether post‐finasteride syndrome “exists,” we may better serve our patients by asking: which individuals are never meant to be prescribed this drug in the first place? This highlights an unmet need: the creation of a brief pre‐treatment psychological screening tool specifically tailored to finasteride candidates. Such a tool could integrate existing measures of health anxiety, body image sensitivity, and nocebo proneness, guiding clinicians in identifying individuals at higher risk of adverse outcomes. The growing availability of finasteride through online platforms—often without in‐person clinical evaluation—further highlights the need for standardized psychological screening, as these systems currently lack tools to detect vulnerable individuals who may be at higher risk of developing persistent side effects. To mitigate the risk of nocebo responses and enhance patient outcomes, I propose the integration of pre‐treatment psychological assessments into the clinical decision‐making process for finasteride therapy. Identifying patients with high levels of health anxiety or those susceptible to nocebo effects can inform tailored counseling strategies [5]. This approach may involve setting realistic expectations, discussing the potential for nocebo responses, and providing reassurance, thereby reducing the likelihood of adverse symptom development attributed to finasteride. In clinical settings, brief validated instruments such as the Whiteley Index [5]—designed to assess health‐related anxiety—and the Body Dysmorphic Disorder Questionnaire (BDDQ) [7]—used to screen for body image disturbance—may serve as practical options. Both tools are self‐administered, time‐efficient, and feasible to implement even in routine outpatient practice. Their use could help identify patients who may benefit from enhanced counseling or psychological referral before initiating finasteride therapy.

Implementing such assessments aligns with a patient‐centered care model, fostering informed decision‐making and potentially decreasing the incidence of treatment discontinuation due to perceived adverse effects. Further research is warranted to develop standardized screening tools and evaluate the efficacy of psychological interventions in this context. In the meantime, integrating brief psychological assessments may represent a simple yet impactful step toward safer and more personalized finasteride prescribing.

## Conflicts of Interest

The author declares no conflicts of interest.

## Data Availability

The author has nothing to report.
